# Subthalamic and Pallidal Deep Brain Stimulation for Parkinson’s Disease

**DOI:** 10.7759/cureus.2232

**Published:** 2018-02-26

**Authors:** Ahmed Negida, Mohamed Elminawy, Gehad El Ashal, Ahmed Essam, Athar Eysa, Mohamed Abd Elalem Aziz

**Affiliations:** 1 Faculty of Medicine, Zagazig University, Egypt, Zagazig University, Egypt; 2 Department of Neurological Surgery, Mayo Clinic, Rochester, MN, USA; 3 Faculty of Medicine, Cairo University; 4 Faculty of Medicine, Beni-Suef University; 5 Faculty of Medicine, Menoufia University; 6 Psychiatry, Amr Shahin Mental Hospital

**Keywords:** deep brain stimulation, parkinson's disease, subthalamic nucleus, globus pallidus internus, motor function

## Abstract

Deep brain stimulation (DBS) is a surgical treatment in which stimulation electrodes are permanently implanted in basal ganglia to treat motor fluctuations and symptoms of Parkinson’s disease (PD). Subthalamic nucleus (STN) and globus pallidus internus (GPi) are the commonly used targets for DBS in PD. Many studies have compared motor and non-motor outcomes of DBS in both targets. However, the selection of PD patients for DBS targets is still poorly studied. Therefore, we performed this narrative review to summarize published studies comparing STN DBS and GPi DBS. GPi DBS is better for patients with problems in speech, mood, or cognition while STN DBS is better from an economic point of view as it allows much reduction in antiparkinson medications and less battery consumption.

## Introduction and background

Parkinson's disease (PD) is a motor disorder characterized by progressive degeneration of dopaminergic neurons in the basal ganglia (BG). This degeneration causes overactivity of subthalamic nucleus (STN) leading to increased globus pallidus internus (GPi) output, which causes PD symptoms. PD is associated with several motor symptoms (rigidity, bradykinesia, postural instability, and tremors) and non-motor symptoms (cognitive problems, dementia, and depression). The etiology of PD is unknown and there is no cure for it. Current symptomatic treatments aim at controlling PD symptoms and improving patients’ quality of life.

Pharmacological treatments enhance the dopaminergic action in the BG. Therefore, they reduce PD symptoms that are mainly due to lack of dopamine. Levodopa (LD) is a widely used pharmacological treatment that induces the conversion of L-DOPA to dopamine by DOPA decarboxylase enzyme in dopaminergic neurons. However, there are two problems with pharmacological treatment: 1) the wearing-off phenomenon in which the duration of action of the drug decreases gradually over time, so patients experience severe PD symptoms between drug intervals, and 2) pharmacological treatment is not effective in all patients; some patients are refractory. Due to the limitations of pharmacological treatments, investigators have developed surgical treatments as pallidotomy, thalamotomy, and deep brain stimulation to overcome the failure of pharmacological treatments.

Deep brain stimulation is a surgical treatment for various neurological disorders. In DBS surgery, electrical stimulation is transmitted to the target site via the implanted electrode. To present, the mechanism of action of DBS is not clear. It is suggested that DBS acts via a depolarization blockade mechanism, releasing local inhibitory neurotransmitters and activating inhibitory neurons that antagonize the effect of dopamine. Therefore, DBS acts through restoring balance in the chemical composition of BG, which decreases motor fluctuations and alleviates PD symptoms. DBS has been widely used because it is reversible and stimulation parameters can be adjusted for each patient by their neurologist. According to Medtronic Inc., about 70,000 patients have undergone DBS surgery between 2002 and 2011 [1]. Subthalamic nucleus (STN) and globus pallidus internus (GPi) are the commonly used targets for DBS in PD.

Many studies have compared motor and non-motor outcomes of DBS in both targets. However, the selection of patients for DBS targets was poorly studied. There is a need to summarize the available evidence to help neurologists select patients for DBS targets. Therefore, we performed this narrative review to summarize published meta-analyses and randomized controlled trials (RCTs) comparing STN DBS and GPi DBS. Table 1 and Table 2 show the design, sample size, and main findings of RCTs comparing STN DBS and GPi DBS. Table 3 summarizes the findings of two meta-analyses directly comparing STN DBS and GPi DBS.

**Table 1:** Summary of design and conclusions of RCTs comparing STN DBS and GPi DBS. RCT= Randomized controlled trial, DBS= Deep brain stimulation, STN= Subthalamic nucleus, GPi= Globus pallidus internus, PD=Parkinson's disease, BDI= Beck depression inventory, UPDRS= Unified Parkinson disease rating scale, Qol= Quality of life, ADL= Activities of daily life, LD= Levodopa.

Study ID and year	Design		Sample size	Intervention	Main findings
George et al. [2] (2014)	Double blind randomized trial	Parallel	37 patients with PD (nine patients were control without DBS)	Bilateral subthalamic or pallidal DBS	STN DBS and GPi DBS had similar effects on the balance and gait of PD subjects. There were some indicators that GPi DBS may be preferable over STN in PD patients with stability concerns.
Odekerken et al. [3] (2013)	RCT	Parallel	128 patients with PD	Bilateral subthalamic or pallidal DBS	There was no significant difference in motor functions between the two groups. GPi DBS reduced dyskinesia more than STN DBS in case of giving the same levodopa equivalent dose (LED). However, STN DBS allowed reduction in LED more than GPi DBS so STN DBS patients might experience less dyskinesia than expected.
Follet et al. [4] (2010)	RCT	Parallel	299 patients with PD	Bilateral subthalamic or pallidal DBS	There was no significant difference in motor function improvement between the two groups at 24 months. Also, no significant difference in Qol between the two groups. Neurocognitive and mood outcomes were better in GPi DBS group than STN DBS group and the authors suggested that STN is a better target for DBS than GPi.
Jones et al. [5] (2010)	RCT	Cross over	12 patients with PD	Two-thirds of participants (8/12) had unilateral DBS and one-third (4/12) had bilateral DBS	There was no statistically significant difference between STN DBS and GPi DBS in terms of speech reaction time.
COMPARE trial [6] (2009)	RCT	Parallel	52 patients with PD	Unilateral subthalamic or pallidal DBS	There was no significant difference between STN DBS and GPi DBS in mood and cognition. STN DBS group showed larger deterioration in verbal fluency scores than GPi DBS especially in the off-medication state and no significant difference in UPDRS motor score between the two groups.
Rothlind et al. [7] (2007)	RCT	parallel	42 patients with PD	Staged bilateral subthalamic or pallidal DBS	STN DBS was associated with small reduction in speed of information processing and memory. Both bilateral and unilateral DBS were associated with small but significant reduction in neuropsychological performance. Declines in semantic verbal fluency were associated with left-sided treatment.
Nakamura et al. [8] (2007)	RCT	Parallel	33 patients with PD	Unilateral subthalamic or pallidal DBS	STN and GPi resulted in similar improvement in hand movements at short term follow-up. Preoperative medication responsiveness predicted improvement in some motor tasks.
Anderson et al. [9] (2005)	RCT	Cross over	23 patients with PD	Bilateral subthalamic or pallidal DBS	UPDRS scores improved; there was no difference between STN DBS and GPi DBS. Bradykinesia improved better in STN DBS group but ADL did not improve by DBS further than medication.
Burchiel et al. [10] (1999)	RCT	Parallel	10 patients with PD	Bilateral subthalamic or pallidal DBS	Both STN DBS and GPi DBS had no significant difference in improvement of UPDRS III motor score during on and off medication conditions. Chronic stimulation of GPi DBS might improve symptoms in combination with LD more than chronic LD/STN stimulation. STN DBS was associated with greater reduction in antiparkinson drugs.

**Table 2:** Summary of design and conclusions of studies on cohort populations from RCTs mentioned in the first table. Summary of design and conclusions of studies on cohort populations from RCTs mentioned in Table 1. RCT= Randomized controlled trial, DBS= Deep brain stimulation, STN= Subthalamic nucleus, GPi= Globus pallidus internus, PD=Parkinson's disease, PIGD= Postural instability gait difficulty, TD= tremor dominant, BDI= Beck depression inventory, UPDRS= Unified Parkinson disease rating scale, Qol= Quality of life, ADL= Activities of daily life, LD= Levodopa, COP= Center of pressure.

Study ID	Design		Sample size	Intervention	Main findings
Katz et al. [11] (2015)	Cohort from multicenter RCT (Follet et al. [4] 2010)	Parallel	235 patients with PD	Bilateral subthalamic or pallidal DBS	There was no significant difference between GPi DBS and STN DBS among different PD motor subtypes. Tremor dominant (TD) motor subtype had significantly greater response to GPi DBS with respect to gait. Postural instability gait difficulty (PIGD) patients obtained the least benefit from both GPi DBS and STN DBS.
Odekerken et al. [12] (2015)	Cohort from (Odekerken et al. [3] 2013)	Parallel	114 patients with PD	Bilateral subthalamic or pallidal DBS	There was no significant difference in neuropsychological outcome between STN DBS and GPI DBS. STN DBS showed greater negative change than GPi DBs in mental speed, attention, and language.
Rothlind et al. [13] (2015)	Cohort from multicenter RCT (Weaver et al. [14] 2009)	Parallel	281 patient with PD	Bilateral subthalamic or pallidal DBS	Few isolated significant neuropsychological changes were detected among STN DBS and GPi DBS groups. There was slightly greater reduction in processing speed after STN DBS than GPi DBS. STN DBS showed a greater reduction in verbal learning and recall performance than GPi DBS.
Weintraub et al. [15] 2013	RCT (Follet et al. [4] 2010)	Parallel	299 patient with PD	Bilateral subthalamic or pallidal DBS	There was no difference between STN DBS and GPi DBS in terms of suicide ideation and behaviors (after 24 months follow-up).
Dietz et al. [16] (2013)	Cohort from COMPARE trial [6]	Parallel	14 patients with PD	Unilateral subthalamic or pallidal DBS	GBi DBS there showed no significant change in verbal fluency performance between different stimulation locations (optimal, ventral, and dorsal) in contrast to (Mikos et al. [17]) findings with STN DBS patients.
Rocchi et al. [18] (2012)	Cohort from multicenter RCT (Weaver et al. [14] 2009)	Parallel	29 Patients with PD	Bilateral subthalamic or pallidal DBS	There was no significant difference in anticipatory postural adjustment (APA) between the two groups.
Weaver et al. [19] (2012)	RCT subset from Weaver et al. [14] 2009)	Parallel	159 patients with PD	Bilateral subthalamic or pallidal DBS	Motor function improvements were similar and statistically significant for both STN DBS and GPi DBS groups at 36 months post surgery but showed decline over time (decline was faster in STN group than GPi group). Qol scale improved at six months but diminished over time. Emotional well-being, social support, and cognition subscales showed no difference over time. Activities of daily living showed early improvement after surgery followed by gradual loss of improvement over the subsequent assessments. Mattis Dementia Rating Scale and the Hopkins Verbal Learning Test scores declined faster for STN than GPi patients by 36 months.
Locke et al. [20] (2011)	Retrospective cohort from COMPARE trial [6]	Parallel	44 patients with PD	Unilateral subthalamic or pallidal DBS	DBS is associated with weight gain with no significant difference between STN DBS and GPi DBS.
Mikos et al. [17] (2011)	Cohort from COMPARE trial [6]	Parallel	17 patients with PD	Unilateral subthalamic or pallidal DBS	STN DBS was associated with decreased letter fluency performance at ventral contacts whereas in optimal contacts STN DBS was associated with improved letter fluency performance.
Robertson et al. [21] (2011)	Cohort from multicenter RCT (Follet et al. [4] 2010)	Parallel	27 patients with PD	Bilateral subthalamic or pallidal DBS	There was a significant improvement in jaw velocity in GPi DBS patients. STN DBS patients had a significantly worse jaw velocity six months after surgery.
Taba et al. [22] (2010)	Cohort from COMPARE trial [6]	Parallel	44 patients with PD	Unilateral subthalamic or pallidal DBS	Patients with GPi DBS were more likely to choose to remain with unilateral implantation. The logistic regression analysis revealed that the odds of proceeding to bilateral DBS were 5.2 times higher for STN DBS than for GPi DBS.
Zahodne et al. [23] (2009)	Cohort from COMPARE trial [6]	Parallel	42 patients with PD	Unilateral subthalamic or pallidal DBS	Unilateral DBS in both STN and GPi improved overall Qol six months after surgery. GPi DBS reported greater improvements in Qol. Verbal fluency problems correlated with poorer Qol on the communication subscale.
Rocchi et al. [24] (2004)	RCT subset from (Burchiel et al. [10] 1999)	Parallel	Nine patients with PD	Bilateral subthalamic or pallidal DBS	Levodopa had less negative side effects on posture in patients with STN DBS than patients with GPi DBS. UPDRS scores were higher in STN DBS patients than GPi DBS in the off condition evident by worse motor signs in STN DBS patients. All center of pressure (CoP) parameters showed deterioration of postural control with DOPA for both the STN DBS and GPi DBS. In on-treatment condition, Cop values were close to normal in STN than GPi. Although, STN groups were more affected by PD. It is suggested that levodopa replacement was more effective for posture in STN DBS group than GPi group.

**Table 3:** Summary of key conclusions of meta-analyses comparing STN DBS and GPi DBS for PD. RCT= Randomized controlled trial, DBS= Deep brain stimulation, STN= Subthalamic nucleus, GPi= Globus pallidus internus, PD=Parkinson's disease, BDI= Beck depression inventory, UPDRS= Unified Parkinson disease rating scale.

Study ID and year	Design	Sample size	Main findings
Sako et al. [25] (2014)	Meta-analysis of four RCTs	patients with PD	There was no significant difference between the two groups in improvement of UPDRS III motor scores. Depression was associated with STN DBS group.
Liu et al. [26] (2014)	Meta-analysis of six RCTs	563 Patients with PD	There was no significant difference between STN DBS and GPi DBS in UPDRS III motor score (on and off medication phases). Activities of daily life on UPDRS II (on medication phase) did not favor either of the two groups. STN DBS was associated with greater reduction in antiparkinson medications. Depression on BDI-II (Beck Depression Inventory) score differed significantly favoring GPi DBS group.
Negida et al. [27] (2015)	Meta-analysis of nine RCTs	497 patients with PD	There was no significant difference between STN DBS and GPi DBS in UPDRS III motor score (on and off medication phases). Activities of daily life on UPDRS II (on medication phase) did not favor either of the two groups. The levodopa equivalent dose was less in patients undergoing STN DBS than GPi DBS. STN DBS allows more reduction in medication than GPi DBS. Subthalmic and pallidal DBS achieved the same motor improvement in PD patients.
Elgebaly et al. [28] (2017)	Meta-analysis of four RCTs	345 patients with PD	There was no statistically significant difference between STN DBS and GPi DBS in neuropsychological outcomes.
Negida et al. [29] (2017)	Meta-analysis of four RCTs	479 patients with PD	Death was more common after STN DBS than GPi DBS in PD patients, most of deaths due to postoperative complications.

## Review

Motor functions and PD motor symptoms

There was no significant difference between STN DBS and GPi DBS in terms of the unified Parkinson's disease rating scale III (UPDRS III) motor score [3, 4, 6, 9-11, 19, 25, 26, 30]. The study of Rocchi et al. [24] was the only study where UPDRS scores were higher in STN DBS patients than in GPi DBS in the off condition evident by worse motor signs in STN DBS patients. In Odekerken et al. [3], STN DBS was associated with improvement in motor symptoms and disability in the off-phase than GPi DBS. However, in Follet et al. [4] and Anderson et al. [9], the GPi DBS group showed more improvement in UPDRS III score and stand-walk-sit times than STN DBS. These better motor scores in GPi DBS were consistent in the 36-month outcome [19]. The higher doses of medication in the GPi DBS group and the increase in stimulation washout time may justify this improvement in the GPi DBS group [4, 19].

In the study of George et al. [2], balance and gait did not differ significantly between the two groups. However, the authors concluded that some predictors make GPi DBS better than STN DBS for patients with stability concerns. In another study, postural instability gait difficulty (PIGD) patients obtained the least benefit from both GPi DBS and STN DBS [11] and tremor dominant (TD) motor subtype showed better response to GPi DBS with respect to gait [11]. Anticipatory postural adjustment (APA) did not differ significantly between the two targets in the study of Rocchi et al. [18]. In another study by Rocchi et al. [24] where they examined postural performance after STN DBS and GPi DBS, they found that levodopa had less negative side effects on posture in patients with STN DBS than GPi DBS. They suggested that levodopa treatment was more effective for posture in STN DBS than in GPi DBS.

Combining medication with stimulation gives better response in GPi DBS while STN DBS gives optimum benefit with much reduction in medication [10, 26]. This suggests a possible synergistic effect between STN DBS and medications [3]. Although some reports described a decline in motor functions over time in case of STN DBS [19, 31, 32], in a large multicenter RCT by Follet et al. [4], there was no decline in motor functions in both STN DBS and GPi DBS groups after 24 months (Table 1).

Activities of daily life and medication dose

Activities of daily life (ADL) did not differ between the two groups in most studies. In unilateral implantation, GPi DBS was associated with higher ADL than STN DBS [6]. A gradual loss in improvement of ADL was noticed in both groups after the 36-month follow-up [19]. When patients received the same levodopa dose to induce the on-medication phase, the STN DBS group experienced more dyskinesia than the GPi DBS group [3]. When given the same levodopa equivalent dose (LED), patients with GPi DBS showed less dyskinesia than patients with STN DBS [3]. However, in the study of Moro et al. [9], STN DBS was likely to improve bradykinesia in the off-medication phase more than GPi DBS. However, most studies showed that STN DBS allowed significant reduction in LED [3, 4, 6, 7, 9, 10, 18, 19, 23, 26] and so they have less dyskinesia. This makes STN a better target for PD patients from an economic point of view [33] and for whom a reduction of medication may be desirable [34].

Quality of life (Qol) improved in patients of both groups at six months but diminished over time. The study of Follet et al. [4], showed no significant difference in Qol between the two targets. But in another study by Zahodne et al. [23], GPi DBS showed better improvement in Qol than STN DBS. However, STN DBS has the advantage of reducing antiparkinsonian medications; this should also be taken into consideration because it may contribute to better quality of life (Qol) in some patients [4] (Table 2).

Verbal fluency

Left lateralized DBS was associated with worsening of the verbal fluency (VF) [7]. STN DBS was associated with worsening of letter verbal fluency after seven months [6]. However, GPi DBS was not associated with verbal fluency problems whatever the stimulation location within globus pallidus was [16]. Verbal fluency was not worsened in STN DBS when low frequency stimulation was used [35]. Getting more ventral in the stimulation location for STN DBS reduced VF [17]. No difference in speech reaction time was noted between both groups [5].

Neuropsychological performance

Self-reported depression was more common with STN DBS than GPi DBS [4, 7, 26]. However, in the 36-month outcome of the study by Follet et al. RCT [4], depression did not differ significantly between the two groups [19]. In the COMPARE trial [6], secondary outcomes revealed increased anger in the STN DBS group only; this anger was reported in previous STN DBS reports [36-38]. Anxiety was fairly common in the STN DBS group in the Anderson et al. study [9]. Visuomotor speed decreased significantly after STN DBS more than GPi DBS [4]. Because digital symbol performance is a task that requires visuomotor coordination, the decline in visuomotor speed after STN DBS justifies the decline in digital symbol performance after STN DBS in the Rothlind et al. RCT [7]. Dementia rating scale worsened more in the STN DBS group than GPi DBS [4, 19]. When Odekerken et al. [3] used a dichotomous composite measure instead of continuous standardized measure for cognition and mood, there was no statistically significant difference between the two groups (Table 2). Elgebaly et al. [28] also showed slight improvement among the GPi DBS group in terms of psychomotor speed and verbal fluency (Table 3).

The relative disturbance in cognition and mood, associated with the STN DBS group, can be justified by the greater possibility of suboptimal lead placement in the STN target. The STN target is smaller in architectural size than the GPi target (~158 mm3 versus ~478 mm3, respectively) [6] and the placement of the lead within STN may correlate with the stimulation of nearby fibers within limbic functions pathways [6, 7]. In the COMPARE trial [6], unilateral implantation did not show a significant difference in cognition and mood between the two targets. Higher baseline neurophysiological performance was associated with more decline in working memory and executive functioning after DBS [7].

Adverse events

Adverse events (AEs) of DBS are classified into five groups: 1) perioperative AEs: headache, anxiety, tension, restless, confusion, and hallucination [39-41]; 2) intraoperative AEs: vasovagal response, syncope, ischemic stroke, hypotension, arrhythmia, confusion, seizure, intracranial hemorrhage, intraventricular hemorrhage, and subdural hematoma [9, 39, 40]; 3) postoperative AEs: symptomatic and asymptomatic intracranial and intraventricular hemorrhage, ischemic infarction, lack of consciousness, and hemiparesis [39, 41]; 4) long term AEs: pain, dysarthria, cognitive dysfunction, paresthesia, and balance disorder [39]; 5) hardware-related complications requiring surgical revision: wound infection, lead migration, lead fracture, and lead malposition [39]. The total number of AEs reported in RCTs comparing STN DBS and GPi DBS was more in STN DBS than in GPi DBS [3, 4, 6, 9]. However, few of them were statistically significant. In the trial of Follet and colleagues [4] fall and depression were common AEs in the STN DBS group. Microelectrode passes were associated with an increased risk of intracranial bleeding [42] (Table 1).

In their meta-analysis, Sako et al. [25] reported that depression was significantly more common in the STN DBS group than in the GPi DBS group while other five AEs were not significant: 1) confusion/delirium, 2) intracranial hemorrhage, 3) dysarthria/speech problems, 4) balance disorder, 5) stroke/transient ischemic attack (TIA). In the other meta-analysis of Liu et al. [26], depression on the Beck Depression Inventory-II (BDI-II) score differs significantly favoring GPi DBS than STN DBS.

It has not escaped our notice that investigators of different studies used different definitions of AEs. There was no standardized definition or outcome measurement for AEs. Therefore, it is difficult to estimate these AEs in meta-analyses models. In their recent meta-analysis of depression and anxiety, Couto et al. [43] concluded that results of relevant clinical trials are heterogeneous. Therefore, standardization of outcome measurement is recommended across centers.

Negida et al. (2017) [29] also concluded death was more common after STN DBS than GPi DBS in PD patients, and most of the deaths were due to postoperative complications (Table 3).

Stimulation amplitude, pulse width, and battery consumption

Stimulation amplitude and pulse width were lower in STN DBS than in GPi DBS, which correlates to low battery consumption [3] and allows longer time intervals between pulse generator replacements. This decreases both the cost and the risk during surgical replacement of pulse generators [4].

## Conclusions

Current evidence suggests that there is no significant difference between STN DBS and GPi DBS in terms of motor improvement on the UPDRS III score although STN DBS allows more reduction in antiparkinsonian medication. STN DBS was associated with more decline in verbal fluency when compared to GPi DBS. Additionally, neuropsychological performance was slightly affected after STN DBS compared to GPi DBS. These findings suggest that the selection of surgical target in PD patients should be done on an individual basis according to patient status and preferences. In patients who are at risk of neuropsychological deterioration and those with speech problems, GPi DBS is a favorable target in order to avoid the possible neuropsychological problems following STN DBS. On the other hand, STN DBS is a favorable surgical target for patients who are not tolerating high doses of levodopa; those patients will take the advantage of medication reductions with STN DBS. In addition, the less battery consumption and the long intervals between pulse generator replacements in case of STN DBS make it a better target from an economic point of view.
